# A diffusion model for mosquito control trials with spillover: application to calculations of power, sample size, and bias

**DOI:** 10.1186/s13063-026-09597-4

**Published:** 2026-03-19

**Authors:** Thomas A. Smith, Luzia N. Felber, Neal A. Alexander, Pélagie E. B. Aboa, Claver N. Adjobi, Julien Z. B. Zahouli, Pie Müller

**Affiliations:** 1 Swiss Tropical and Public Health Institute, Kreuzstrasse 2, Allschwil, 4123 Switzerland; 2 University of Basel, Basel, 4001 Switzerland; 3 MRC International Statistics and Epidemiology Group, London School of Hygiene and Tropical Medicine, London, UK; 4 Centre d’Entomologie Médicale Et Vétérinaire de L’Université Alassane Ouattara, Bouaké, Côte d’Ivoire; 5 Centre Suisse de Recherches Scientifiques en Côte d’Ivoire, Abidjan, Côte d’Ivoire; 6 Université Félix Houphouët-Boigny, Abidjan, Côte d’Ivoire

**Keywords:** Cluster, Diffusion, Movement, Power bias, Mosquito, Aedes, Anopheles

## Abstract

**Background:**

Mosquito movement is ubiquitous and complicates the design of cluster randomized trials (CRTs) of vector control interventions because it can cause spillover between trial arms. There has been no analytical approach to quantify the impact of this spillover on trial power, required sample sizes, or bias. This precludes formal allowance for spillover in formulae for study power, and a precautionary principle is generally used in trial design, leading to CRTs with very large clusters and extensive buffer zones between arms.

**Methods:**

The diffusion equation was solved to give a mathematical model for mosquito displacement in a cluster randomized trial. This provides an explicit function for calculating the bias in efficacy estimates due to mosquito movement. Substituting this into a conventional formula for the power gives adjusted sample size and power estimates and indicates how the exclusion of data from buffer zones affects bias and power. The method is generally applicable to trials with entomological outcomes, or to malaria trials with epidemiological outcomes, providing these can be related to biting densities of mosquitoes. It is illustrated with the baseline data from an intervention trial against *Aedes aegypti* in Côte d’Ivoire.

**Results:**

Despite the small size of the Côte d’Ivoire trial, it has adequate power to detect true intervention efficacy of ≥0.4, even in the presence of considerable spillover. If the spillover extends over a wide distance, the power will be considerably increased by using buffer zones.

**Conclusions:**

The analytical approach can be incorporated into power calculations for CRTs with geographical spillover, whenever the diffusion model is considered an acceptable approximation. The example suggests that the key to obtaining adequate power in the presence of spillover is the inclusion of a sufficiently large number of clusters, even if only a small amount of data can be obtained from each cluster. The method can be implemented in a publicly available R package.

## Background

Mosquito-borne diseases transmitted by Aedes and Anopheles account for about 17% of all infectious disease, causing more than 700,000 deaths annually [1] and there is a continual need to discover and trial new preventive interventions that interfere with the mosquito life cycle. Mosquito movement is a complication in such trials.

Randomized trials of interventions such as indoor residual spraying or the distribution of mosquito nets make use of geographical clusters composed of groups of adjacent households. One reason for this clustering is that the mosquito movement leads to spillover (contamination) which reduces the measured efficacy if measurements in the opposite trial arms are close to each other. (Here we use spillover and contamination as synonyms, although some authors use the latter term to refer specifically to receipt of an intervention (e.g. bed nets) to which they were not allocated [2, 3]). Trials of such interventions are generally designed with either very large clusters, and/or with buffer zones between the clusters. Both these procedures ensure that only a small proportion of the measurements are taken close to locations in the opposite arm of the trial. This minimizes spillover, but at the cost of making trials very large. Existing formulae for sample size and power calculations for CRTs do not allow for effects of spillover, so trial designs and power calculations for such cluster randomized trials (CRTs), with or without buffers, generally neglect any residual spillover and its effect in biasing the outcome.

Mosquito movement causes spillover because of the displacement between where interventions act and where the outcome is measured. On average the mean distance travelled for *Aedes aegypti* mosquitoes is of the order of 100 m [4]. Anopheles mosquitoes travel further, but the data on this are sparse, and the distribution of how far they fly is highly uncertain. In a landscape with few target sites the optimal strategy for an organism searching for prey or other resources can be described by Lévy distributions [5], but if there are many approximately homogeneously distributed olfactory or visual stimuli the central limit theorem argues that distributions of displacement distances should approximate normality. This is equivalent to a diffusion model for movement of the searching organism. In a CRT, the availability of breeding sites and exposed humans and various other landscape heterogeneities may all affect mosquito movement, so if there are many clusters the diffusion model should provide a good approximation. This model also provides a reference against which other movement models might be evaluated.

This paper uses the diffusion model of mosquito movement to predict the bias resulting from spillover in the estimate of efficacy in a CRT. The model is based on solving the heat- or diffusion equation used in thermodynamics, making use of standard results for normal integrals [6]. Power and the required sample size are calculated by substituting the biased effect size into a conventional sample size formula. A trial designed this way is powered to estimate the biased efficacy via a conventional data analysis. Efficacy estimates made this way can be adjusted upwards to allow for the spillover [7].

The approach is illustrated with power calculations from the baseline dataset of an intervention trial against *Aedes aegypti* in Côte d’Ivoire.

## Methods

### Model for mosquito movement

A simple mathematical model for a mosquito population that is homogeneous in space and time is that mosquitoes emerge at rate $\lambda$ and die at rate $\mu$, so that the steady state density is ${u}_{0}=\lambda /\mu$, obtained as the solution of the birth–death process:

1 $$\frac{du}{dt}=\lambda -u\mu .$$

A parsimonious way to extend population models to include mosquito movement is to treat it as equivalent to diffusion [8, 9]. Ignoring any directional effects, such as prevailing winds, this is an isotropic process described by the diffusion equation. In the two-dimensional case (ignoring vertical movement) and using Cartesian coordinates, the mosquito density ${u}_{1}\left(x,y, t\right)$ at location $\left(x,y\right)$, time *t*, arising from dispersion from a single point source of ${u}_{1}\left(\mathrm{0,0},0\right)$ mosquitoes at $x=y=0$, is then the solution of the diffusion/heat equation:

2 $$\frac{\partial u}{\partial t}=D\left(\frac{{\partial }^{2}u}{\partial {x}^{2}}+ \frac{{\partial }^{2}u}{\partial {y}^{2}}\right),$$

with boundary conditions ${u}_{1}\left(x,y,t\right)\to 0, x \to \pm \infty$ and ${u}_{1}\left(x, y,t\right)\to 0, y \to \pm \infty$, which is [9]:

3 $${u}_{1}\left(x,y,t\right)= \frac{1}{4\pi Dt}exp\left(\frac{-\left({x}^{2}{+ y}^{2}\right)}{4Dt}\right),$$

where $D$ is the diffusion coefficient (assumed constant) (Table 1).

**Table 1:** Quantities in models of movement and bias

Symbol	Quantity	Dimension
$t$	Time	Time
$u$	Mosquito density	Relative number of mosquitoes
$\lambda$	Emergence rate of mosquitoes	Relative number of mosquitoes $\times$ time^−1^
$\mu$	Mortality rate of mosquitoes	Relative number of mosquitoes $\times$ time^−1^
$x$	Distance from boundary between trial arms	Signed distance
$y$	Distance perpendicular to boundary	Distance
$D$	Diffusion coefficient	Distance^2^ $\times$ Time^−1^
$E$	Trial efficacy	Dimensionless
$I(x)$	Indicator of trial arm	Heaviside step function
$\Phi ()$	Standard normal cumulative distribution function (cdf)	Function
$\phi ()$	Standard normal probability density (pdf)	Probability density
$Q\left(z,\sigma \right)$	Quantile of normal cdf with s.d. $\sigma$ and mean zero	Dimensionless
$p\left(x\right)$	Generic probability density	Probability density
$C$	Constant arising in differentiation	Relative number of mosquitoes
$\sigma ()$	Mean mosquito displacement	Distance
${\sigma }_{m}$	Mean mosquito displacement in unit time	Distance
$s$	Spillover interval = $2\Phi (0.975){\sigma }_{m}$	Distance
${\sigma }_{x}$	Standard deviation of $x$	Distance
${\sigma }_{r}$	Ratio of ${\sigma }_{m}/{\sigma }_{x}$	Dimensionless
$\widetilde{E}$	Expectation of naïve efficacy estimate	Dimensionless
$\delta$	Bias in naïve efficacy estimate	Dimensionless
${P}_{s}$	Proportion of locations in spillover interval	Dimensionless

**Fig. 1:**
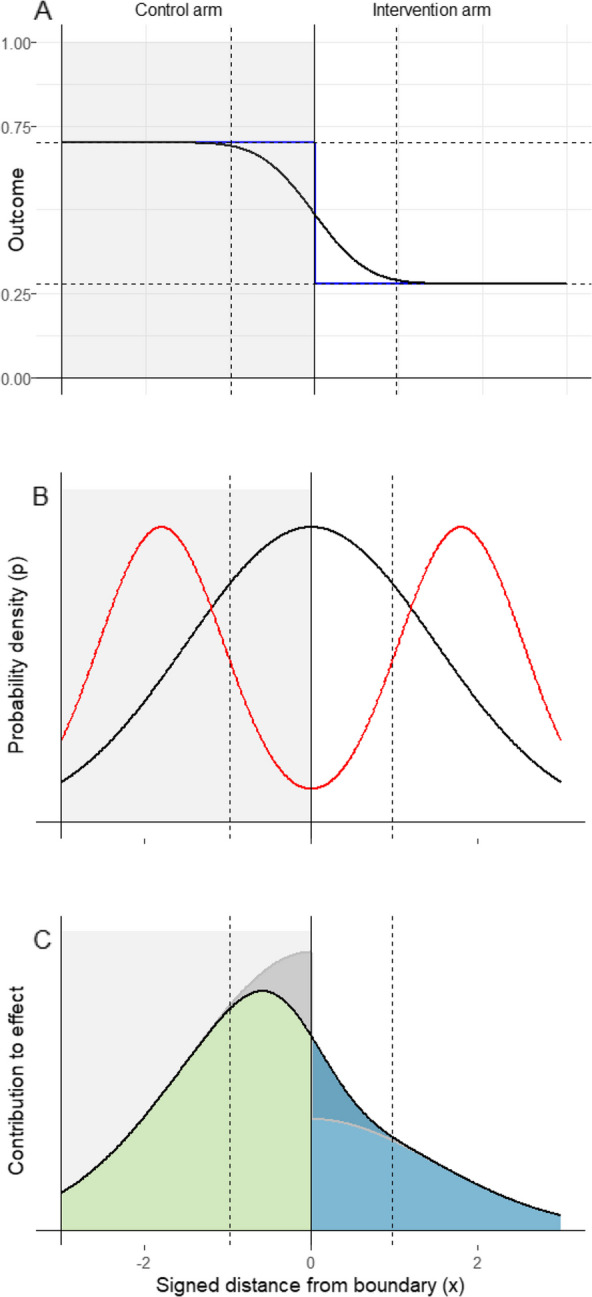
Schematics illustrating calculation of bias. The grey shading corresponds to the control zone; the vertical dashed lines indicate the spillover interval within which 95% of the spillover occurs, at ${\upsigma }_{\mathrm{m}}=0.5.$** A** Expected outcome, $\mathrm{u}\left(\mathrm{x}\right)$, as a function of distance. Blue line: without spillover (Eq. (13)); black line: with spillover (Eq. (11)). **B** Probability densities by distance. Black line: $\mathrm{p}\left(\mathrm{x}\right)=\frac{1}{{\upsigma }_{\mathrm{m}}}\upphi \left(\frac{\mathrm{x}}{{\upsigma }_{\mathrm{m}}}\right)$ Red curve: $\mathrm{p}\left(\mathrm{x}\right)$ for a trial with well-separated clusters (excess kurtosis of −1.4). **C** Black line: ${{u}}\left({{x}}\right){{p}}\left({{x}}\right)$ for the normal case from panel **B**. The coloured areas correspond to the functions that are integrated in Eq. (15), blue: ${\int }_{0}^{{\infty }}\left(1-{{E}}{\Phi }\left(\frac{{{x}}}{{{{\sigma}}}_{{{m}}}}\right)\right){{p}}\left({{x}}\right){{d}}{{x}}$, and green: ${\int }_{-{\infty }}^{0}\left(1-{{E}}{\Phi }\left(\frac{{{x}}}{{{{\sigma}}}_{{{m}}}}\right)\right){{p}}\left({{x}}\right){{d}}{{x}}$. The dark shaded areas correspond to the effect of spillover

In the two-patch model for a CRT, where $x$ is the Euclidean distance to the boundary between arms, (with the control zones are specified by $x<0$, and the intervention zone as $x\ge 0$) the efficacy, defined as the effect size induced at location $\left(x, y\right)$ is a step function of $x$,$EI\left(x\right)$ where $I\left(x\right)$ is an indicator of the sign of $x$ (Fig. 1A). In this system, $u\left(x, y,t\right)$ is independent of $y$, so that in the presence of spillover approximated with diffusion, the mosquito density, $u\left(x,t\right),$ is a solution of the one- dimensional diffusion equation:

4 $$\frac{\partial u}{\partial t}=D\frac{{\partial }^{2}u}{\partial {x}^{2}},$$

with boundary conditions $u\left(x,t\right)\to \lambda /\mu , x \to -\infty$; $u\left(x,t\right)\to \lambda \left(1-E\right)/\mu , x \to +\infty$; and $u\left(x,t\right)=\lambda \left(1-EI\left(x\right)\right)/\mu$, for $t=0$. An expression for $u\left(x,t\right)$ is obtained from the convolution of the initial condition $g\left(x\right)= u\left(x,0\right)$ with the solution of the one-dimensional diffusion/heat equation, ${u}_{2}\left(x,t\right)= \frac{1}{\sqrt{4\pi Dt}}exp\left(\frac{-{x}^{2}}{4Dt}\right)$, which is [10]:

5 $$\begin{array}{c}u\left(x,t\right)={\int }_{-\infty }^{\infty }{u}_{2}\left(x-\zeta ,t\right) g\left(\zeta \right) d\zeta\\=\frac\lambda\mu\int_{-\infty}^\infty u_2\left(x-\zeta,t\right)d\zeta-C\int_\mathrm{x}^{-\infty}u_2\left(\xi,t\right)\left(-1\right)d\xi,\end{array}$$

with $C=\lambda E/\mu$. This yields a candidate for a solution of the form:

6 $$u\left(x,t\right)=\frac{\lambda }{\mu }\left(1-E\Phi \left(\frac{x}{\sigma \left(t\right)}\right)\right),$$

where $\Phi (\frac{x}{\sigma \left(t\right)})$ is the cumulative function of the normal distribution with mean zero and standard deviation $\sigma \left(t\right)=\sqrt{2Dt}$, which varies with time.

Differentiation with respect to $x$ gives:

7 $$\frac{\partial u}{\partial x}= \frac{-C}{\sigma \left(t\right)}\phi \left(\frac{x}{\sigma \left(t\right)}\right),$$

where $\phi (x)$ is the standard normal probability density function, and:

8 $$\frac{{\partial }^{2}u}{{\partial x}^{2}}=\frac{Cx}{{\left(\sigma \left(t\right)\right)}^{3}}\phi \left(\frac{x}{\sigma \left(t\right)}\right).$$

Differentiation with respect to $t$ gives:

9 $$\frac{\partial u}{\partial t}= \frac{Cx}{{\left(\sigma \left(t\right)\right)}^{2}}\phi \left(\frac{x}{\sigma \left(t\right)}\right)\frac{d\sigma }{dt} .$$

Therefore, the diffusion equation is satisfied if:

10 $$\begin{array}{c}\frac{Cx}{{\left(\sigma \left(t\right)\right)}^{2}}\phi \left(\frac{x}{\sigma \left(t\right)}\right)\frac{\partial \sigma }{\partial t}=\frac{DCx}{{\left(\sigma \left(t\right)\right)}^{3}}\phi \left(\frac{x}{\sigma \left(t\right)}\right)\\ \frac{\partial \sigma }{\partial t}=\frac{D}{\sigma \left(t\right)}\\ \sigma \left(t\right)=\sqrt{2Dt}.\end{array}$$

It follows that the diffusion model for spillover in the CRT, satisfying the boundary conditions, is:

11 $$u\left(x,t\right)=\lambda /\mu \left(1-E\Phi \left(\frac{x}{{\sigma }_{m}}\right)\right),$$

where ${\sigma }_{m}=\sqrt{2Dt}$ is the standard deviation of the cumulative normal representing the displacement of a mosquito during the time interval between exposure to the intervention and measurement of the effect (Table 1). In typical applications, this time interval is equal to the duration of the gonotrophic cycle, i.e. the time interval between successive attempts to bite human hosts.

### Model for bias in efficacy estimates

In the general case, the expected value of the measured efficacy is:

12 $$E=1-\frac{{\int }_{0}^{\infty }u\left(x\right)p\left(x\right)dx}{{\int }_{-\infty }^{0}u\left(x\right)p\left(x\right)dx},$$

where $u\left(x\right)$ is the expected value of the outcome at the time of measurement and $p\left(x\right)$ is the probability density of $x$ with mean zero, which is a function both of cluster geometry and the density in space of the sampled points (Fig. 1). In the absence of spillover, the effects are measured at the same place as they are induced and the event rates in the control and intervention arms are ${\lambda }_{0}={\int }_{-\infty }^{0}u\left(x\right)p\left(x\right)dx$ and ${\lambda }_{1}={\int }_{0}^{\infty }u\left(x\right)p\left(x\right)dx,$ respectively, so the efficacy is:

13 $$E=1-\frac{{\lambda }_{1}}{{\lambda }_{0}},$$

corresponding to an expected outcome as a function of distance of $u\left(x\right)=\lambda \left(1-EI\left(x\right)\right)/\mu$. If other sources of bias can be discounted, an unbiased estimate of efficacy may be obtained from the usual formula:

14 $$\widehat{E}=1-\frac{{\overline{u} }_{\mathrm{I}}}{{\overline{u} }_{\mathrm{C}}},$$

where ${\overline{u} }_{C}$ is the mean of the outcome in the control arm, and ${\overline{u} }_{I}$ is the mean of the outcome in the intervention arm.

In the presence of mosquito movement Eq. (11) gives the expected value of the outcome and the naïve estimate of efficacy from Eq. (14) then has expected value:

15 $$\widetilde{E}= 1-\frac{{\int }_{0}^{\infty }{u}_{S}\left(x\right) p\left(x\right) dx}{{\int }_{-\infty }^{0}{u}_{S}\left(x\right) p\left(x\right) dx}=1-\frac{{\int }_{0}^{\infty }\left(1-E\Phi \left(\frac{x}{{\sigma }_{m}}\right)\right)p\left(x\right)dx}{{\int }_{-\infty }^{0}\left(1-E\Phi \left(\frac{x}{{\sigma }_{m}}\right)\right)p\left(x\right)dx},$$

where ${u}_{S}\left(x\right)$ is the outcome as modified by the effect of spillover, depending on ${\sigma }_{m}$. $\delta =\widetilde{E}-E$ measures the effect of spillover (Fig. 1B) and is here referred to as the spillover bias. Spillover reduces estimated efficacy and hence $\delta$ has a negative sign.

Because of the randomization, $p\left(x\right)$ should approximate a symmetric distribution with mean zero. A reasonable proposal is a normal distribution with standard deviation ${\sigma }_{x}$, so that $p\left(x\right)=\frac{1}{{\sigma }_{x}}\phi \left(\frac{x}{{\sigma }_{x}}\right)$ (see example) and $\delta$ is then:

16 $$\begin{array}{c}\delta =\widetilde{E}-E=1-\frac{{\int }_{0}^{\infty }\left(1-E\Phi \left(\frac{x}{{\sigma }_{m}}\right)\right)\frac{1}{{\sigma }_{x}}\phi \left(\frac{x}{{\sigma }_{x}}\right)dx}{{\int }_{-\infty }^{0}\left(1-E\Phi \left(\frac{x}{{\sigma }_{m}}\right)\right)\frac{1}{{\sigma }_{x}}\phi \left(\frac{x}{{\sigma }_{x}}\right)dx}-E,\\=1-\frac{\int_0^\infty\left(1-E\Phi\left(\frac v{\sigma_r}\right)\right)\phi\left(v\right)dv}{\int_{-\infty}^0\left(1-E\Phi\left(\frac v{\sigma_r}\right)\right)\phi\left(v\right)dv}-E,\end{array}$$

where ${\sigma }_{r}={\sigma }_{m}/{\sigma }_{x}$ and $v$ is substituted for $x/{\sigma }_{x}.$

Standard integration results [5] are:

17 $$\begin{array}{c}{\int }_{0}^{\infty }\Phi \left(\frac{v}{{\sigma }_{r}}\right)\phi \left(v\right)dv= \frac{1}{2\pi }\left(\frac{\pi }{2}+ arctan\left(\frac{1}{{\sigma }_{r}}\right)\right)\\ {\int }_{-\infty }^{0}\Phi \left(\frac{v}{{\sigma }_{r}}\right)\phi \left(v\right)dv= \frac{1}{2\pi }\left(\frac{\pi }{2}- arctan\left(\frac{1}{{\sigma }_{r}}\right)\right).\end{array}$$

So, an explicit expression for $\delta$ in the normal case is:

18 $$\delta =1-\frac{1- E\left(\frac{1}{2}+\frac{1}{\pi } arctan\left(\frac{1}{{\sigma }_{r}}\right)\right)}{1- E\left(\frac{1}{2}-\frac{1}{\pi } arctan\left(\frac{1}{{\sigma }_{r}}\right)\right)}-E.$$

Equivalently, $\delta$ can be expressed as a function of $E$ and the quantiles of $\phi \left(\frac{x}{{\sigma }_{m}}\right).$ Defining ${P}_{s}$ as the proportion of data falling within the spillover interval (Table 1) and hence satisfying ${\Phi }^{-1}\left(0.025\right)<\frac{x}{{\sigma }_{m}}< {\Phi }^{-1}\left(0.975\right)$, we get:

19 $${P}_{s}= {\int }_{-z{\sigma }_{m}}^{z{\sigma }_{m}}p\left(x\right)dx={\int }_{-z{\sigma }_{r}}^{z{\sigma }_{r}}\phi \left(w\right) dw=1-2\Phi \left(-{z\sigma }_{r}\right),$$

where $z={\Phi }^{-1}\left(0.975\right) \approx 1.96$ and $w=x/{\sigma }_{x}$. It follows that:

20 $${\sigma }_{r}=\frac{{\Phi }^{-1}\left(\left(1-{P}_{s}\right)/2\right)}{{\Phi }^{-1}\left(0.025\right)}.$$

The value of ${\sigma }_{r}$ can be substituted in Eq. (18) to obtain $\delta$*.*

### Power calculations

The power calculations are applicable to a data analysis that does not include any adjustment for the spillover, for instance using cluster-level summaries of the data and comparing means between arms via a *t*-test [11]. Standard calculations for the two-armed CRT without spillover [11] use the formula:

21 $${z}_{1-\beta }=\sqrt{\frac{\left(\mathrm{c}-1\right){\left({\lambda }_{0}-{\lambda }_{1}\right)}^{2}}{\left({\lambda }_{0}+{\lambda }_{1}\right)/\Upsilon +\left({k}_{0}^{2}{\lambda }_{0}^{2}+{{k}_{1}^{2}\lambda }_{1}^{2}\right)}}-{z}_{1-\alpha /2}.$$

where the different quantities are as given in Table 2, and the power is $\Phi \left({z}_{1-\beta }\right)$.

**Table 2:** Additional quantities in power calculations

Symbol	Quantity
$c$	Number of clusters in each arm
${\lambda }_{0}$	Event rate in control arm
${\lambda }_{1}$	Event rate in intervention arm
$\Upsilon$	Denominator per cluster
${\delta }_{t}$	Bias in event rate
$k$	Coefficient of variation in event rate

In the case with symmetric spillover, the event rates in the presence and absence of intervention are biased by amounts of equal magnitude and opposite sign, $-{\delta }_{t}/2$ and ${\delta }_{t}/2,$ respectively, so that:

22 $$\widetilde{E}=E+\delta =1-\frac{{\lambda }_{1}}{{\lambda }_{0}}+\delta =1-\frac{{\lambda }_{1}-{\delta }_{t}/2}{{\lambda }_{0}+{\delta }_{t}/2}.$$

and:

23 $${\delta }_{t}=\frac{2\delta {\lambda }_{0}^{2}}{{\lambda }_{1}-\delta {\lambda }_{0}+{\lambda }_{0}},$$

(which, like $\delta$, is negative for all cases with spillover). Replacing ${\lambda }_{1}$ with ${\lambda }_{1}-{\delta }_{t}/2$ and ${\lambda }_{0}$ with ${\lambda }_{0}+{\delta }_{t}/2$ in Eq. (21) leads to:

24 $${z}_{1-\beta }=\sqrt{\frac{\left(\mathrm{c}-1\right){\left({\lambda }_{0}-{\lambda }_{1}+{\delta }_{t}\right)}^{2}}{\left({\lambda }_{0}+{\lambda }_{1}\right)/\Upsilon +\left({k}_{0}^{2}{\left({\lambda }_{0}-{\delta }_{t}/2 \right)}^{2}+{k}_{1}^{2}{\left({\lambda }_{1}+{\delta }_{t}/2 \right)}^{2}\right)}}-{z}_{1-\alpha /2}.$$

If the coefficient of variation, $k$, is the same in both arms this simplifies to:

25 $${z}_{1-\beta }=\sqrt{\frac{\left(\mathrm{c}-1\right){\left({\lambda }_{0}-{\lambda }_{1}+{\delta }_{t}\right)}^{2}}{\left({\lambda }_{0}+{\lambda }_{1}\right)/\Upsilon +{k}^{2}\left({\left({\lambda }_{0}-{\delta }_{t}/2 \right)}^{2}+{\left({\lambda }_{1}+{\delta }_{t}/2 \right)}^{2} \right)}}-{z}_{1-\alpha /2}.$$

Substitution of ${\delta }_{t}$ from Eq. (23) into Eqs. (24) and (25), using a value of $\delta$ obtained from Eq. (18) or Eq. (20) thus gives the power of a CRT for any input value of ${\sigma }_{r}$, or if ${\sigma }_{x}$ is known, for any value of ${\sigma }_{m}$, conditional on the other quantities in Eqs. (24) or (25).

**Fig. 2:**
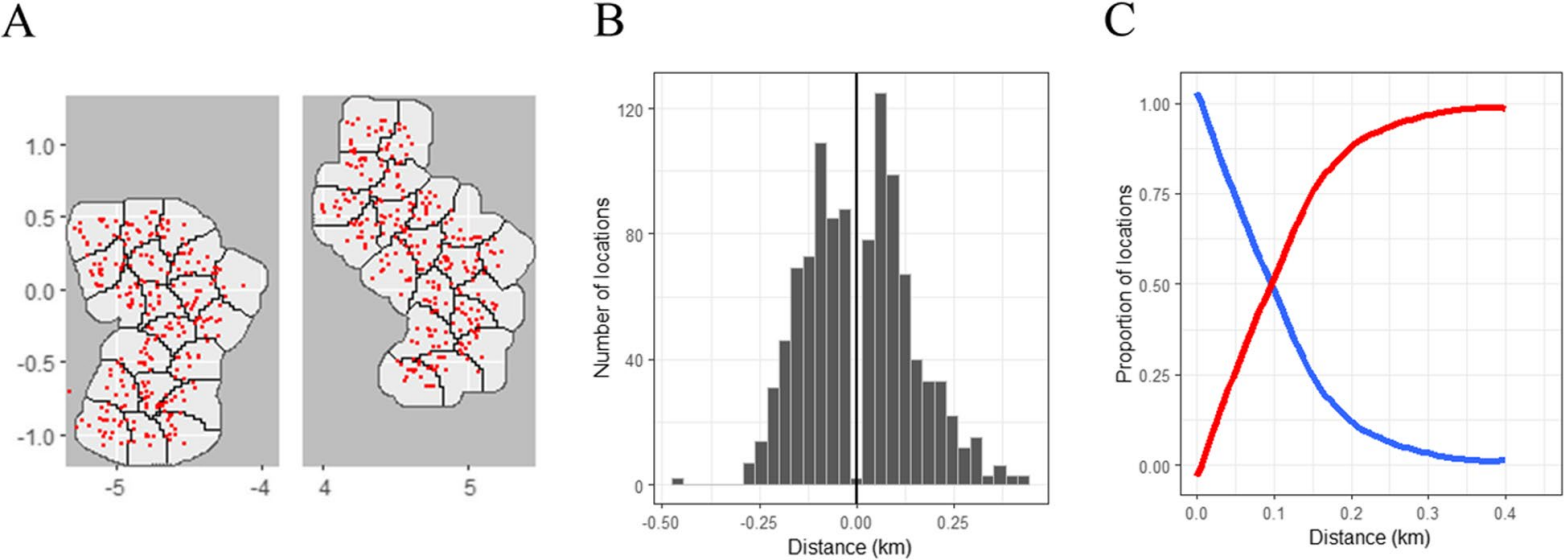
Distances to nearest discordant pixel. **A** Map of study area showing cluster boundaries and sampled locations in red (units are km). **B** Histogram of signed distance to nearest discordant pixel. **C** Blue: proportion of locations in core by buffer width; red: proportion in spillover interval, ${{\boldsymbol{P}}}_{{\boldsymbol{s}}}$, by interval half width (${\boldsymbol{s}}/2$)

Alternatively, if the sampled locations are available (for instance from a baseline survey) then the empirical distribution can be used for $p\left(x\right)$ to obtain $\delta =\widetilde{E}-E$ from Eq. (15). In general, clusters should be specified in a way that reduces $p\left(x\right)$ for small absolute values of $x$, as in the red curve illustrated in Fig. 1B. The effect of this on $\delta$ and power ($1-\beta$) is illustrated in Fig. 2.

### Example

The example dataset is the baseline from a trial of community-based interventions (larval source management and mosquito trapping) against *Aedes aegypti* in the Anono and Gbagba neighbourhoods of Abidjan, Côte d’Ivoire [12].

The study site was divided into 40 equal area clusters, 20 in each of the neighbourhoods (Fig. 2A). The clusters were based on a grid of 4000 pixels, each of area 625 m^2^, making a total area of approximately 2.5 km^2^. Sixteen weeks of sampling of mosquitoes were planned, with sampling on two consecutive days from dawn to dusk (i.e. 06.00–18.00 h) per cluster per week. Mosquito collectors were provided with target locations to sample on each occasion, but for practical reasons could not sample exactly at the chosen points. The target points were randomly sampled from the centroids of the pixels, excluding the periphery of each cluster, thus reducing erroneous sampling of the adjacent cluster.

The example dataset was analysed using R Statistical Software versions 4.3.1 [13]; 1064 out of the target of 1280 samples could be analysed. The remainder were excluded owing to technical failures of the traps or invalid data. The sampled locations were assigned to clusters according to the nearest gridded pixel which was not always the cluster of the target location because fieldworkers’ searches for suitable locations to place a trap sometimes took themselves into another pixel. Some locations were sampled several times, with a total of 531 distinct locations sampled, a mean number of samples per location of 2.0 and a per cluster mean number of locations of 13.3. Most, but not all, sampled points fell within the grid (Table 3). The sampled points tended to be nearer to the centroids of the clusters than were the centroids of the pixels making up the grid.

**Table 3:** Distributions of distances in the example dataset (m)

	**Min**	**Q1**	**Median**	**Mean**	**Q3**	**Max**
Distance of sampled points to the margin of the nearest pixel*	−13.9	−7.4	−4.1	−3.1	−1.5	41.8
Distance of sampled points to the centroid of the cluster	10.2	63.9	90.8	93.5	118.8	561.7
Distance of centroids of the pixels to the centroid of the cluster	1.1	72.9	103.0	103.1	131.7	272.0

*A circular approximation was used for the margin of the pixel. For points within the pixel, the distance to the margin is assigned a negative sign

A mean of 3.4 mosquitoes was captured at each sampled location, over all days of trapping. A Poisson model fitted by GEE was used to estimate the coefficients of variation for the inter-cluster variation from the data (aggregated at the level of the location). This gave an estimate of mosquitoes caught per trap-day of 1.69 (95% CL: 1.53, 1.86) and of the coefficient of variation between clusters of 34.1% (95% CL: 27.4, 45.4). 

Analyses of expected spillover, bias, and power assume that the intervention phase of the trial will compile a database comparable in size to that from the baseline. For each sampled point, the distance, $x$, to the margin of the opposite (discordant) arm of the trial was computed. The distribution of $x$ is bimodal (Fig. 2B), as with the red curve in Fig. 1B. The proportion of locations with $|x|<\widetilde{x}$ strongly decreases with $\widetilde{x}$ (Fig. 2C), so that the proportion in the core is already low with a buffer of 200 m. This corresponds to a value close to unity for ${P}_{s}$, the proportion of locations in a spillover interval $s$ = 400 m.

**Fig. 3:**
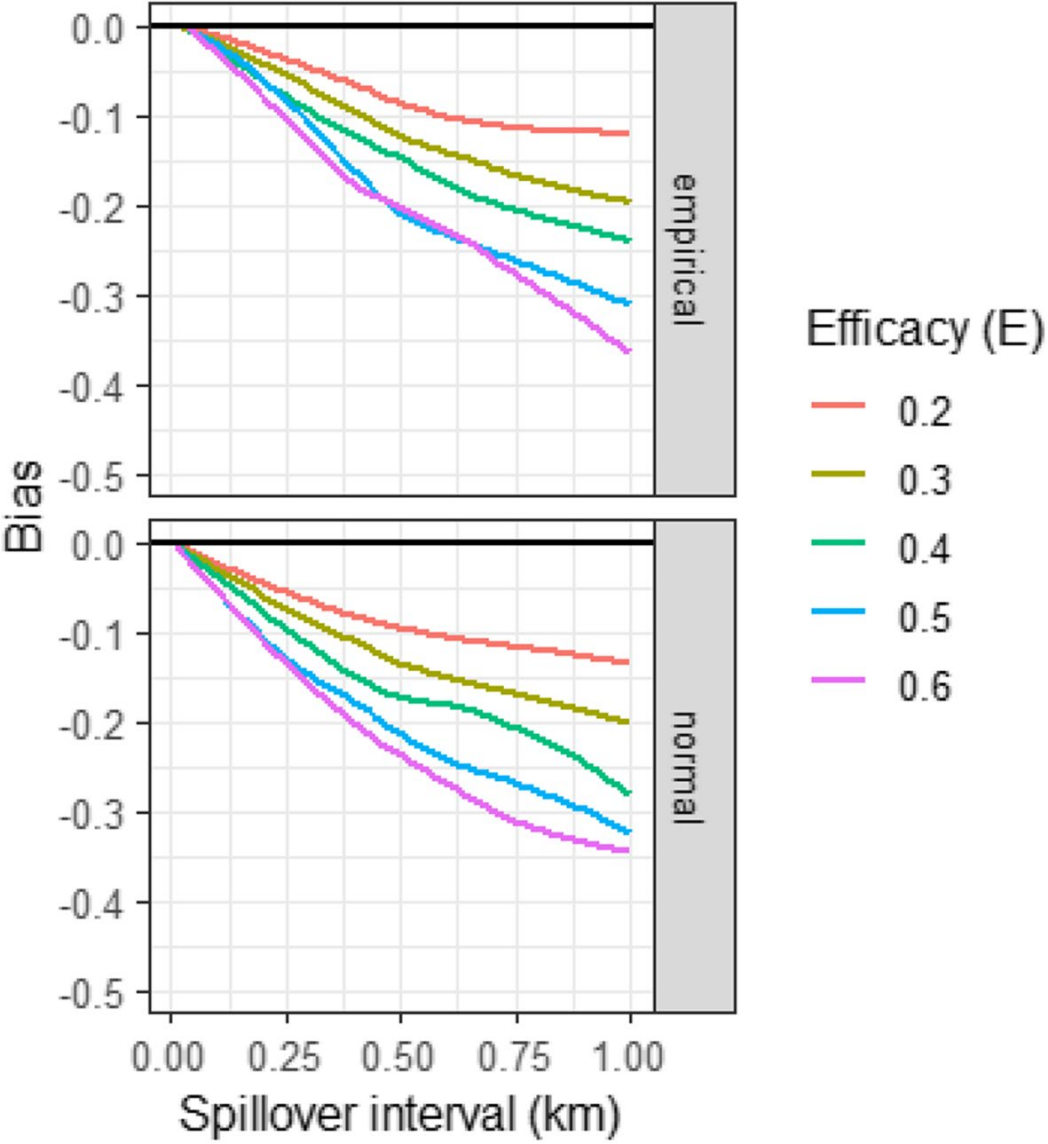
Bias by efficacy and spillover interval. The horizontal lines correspond to no bias. The panels correspond to whether the bias was estimated using Eq. (15) (empirical) or Eq. (18) (normal)

Since there are no local data on mosquito movement from which to estimate the spillover interval, $s$, a sensitivity analysis was used to analyse how the predicted $\delta$ varies with both, $s$, and the efficacy of the intervention, $E$ (Fig. 3), using either the empirical distribution of $p(x)$ (Eq. (15)) or the normal approximation (Eq. (18)) implemented in the CRTspat R package [14] (https://thomasasmith.github.io/reference/CRTpower.html). The curves do not have any parametric shape because they depend on the exact co-ordinates of the sampled locations; however, $\delta$ decreases approximately linearly with $s$, corresponding to a bias of greater magnitude in. The empirical distribution of distances gives slightly less bias than the normal approximation, but the differences are very small, despite the clear deviation from normality evident in Fig. 2B.

The power of the trial, allowing for $\delta$, was obtained from Eq. (25). The calculated power for different spillover intervals and different true effect sizes shown in Fig.

**Fig. 4:**
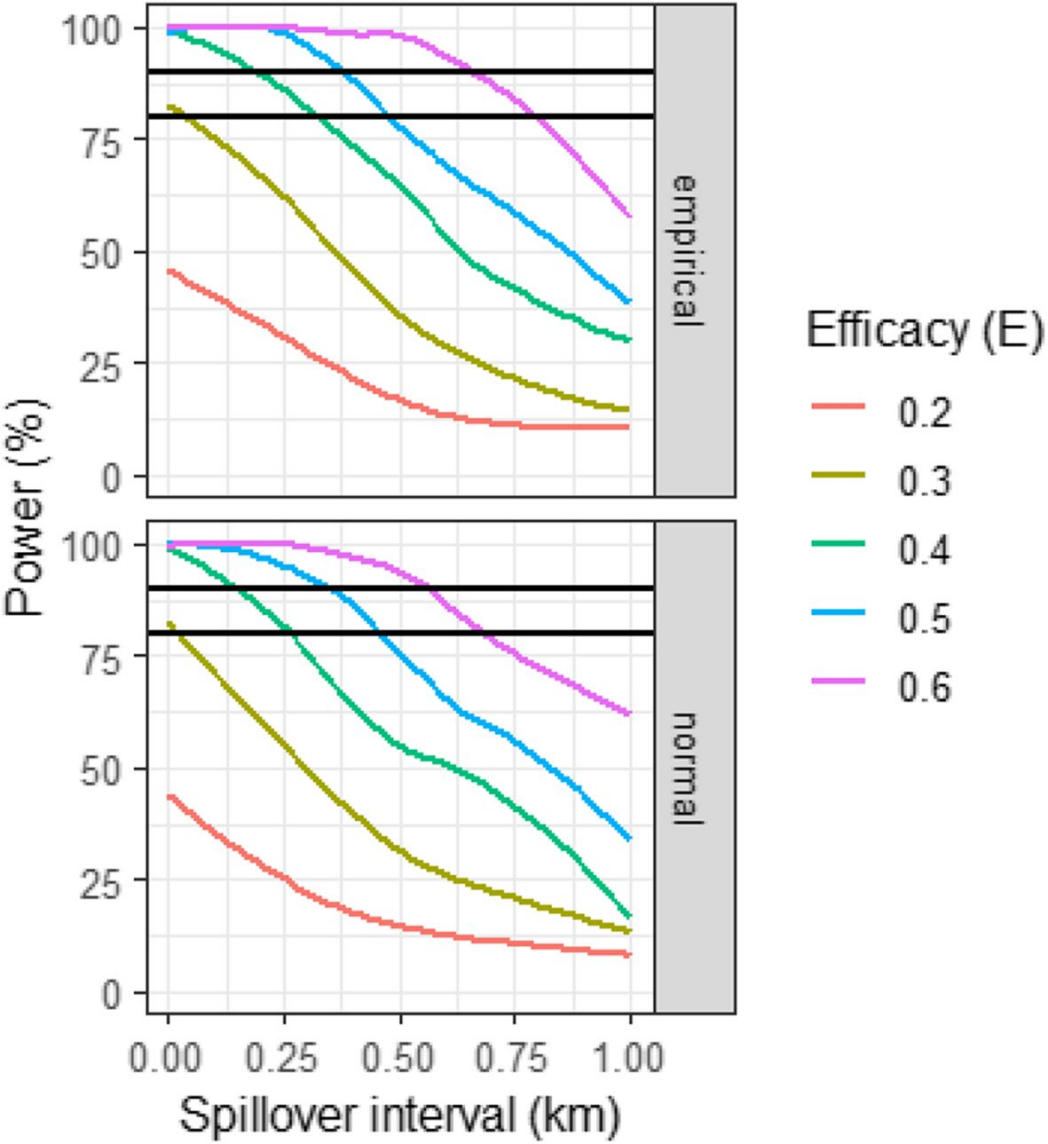
Power by efficacy and spillover interval. The horizontal lines correspond to the conventional thresholds of 80% and 90%. The panels correspond to whether the bias was estimated using Eq. (15) (empirical) or Eq. (18) (normal)

4 shows a similar pattern to that seen for $\delta$. Acceptable (>80%) power is observed for effect sizes of 0.4 or above, at plausible values of the spillover interval. The use of the empirical distribution of distances again gives a slightly less conservative value than does the normal approximation.

**Fig. 5:**
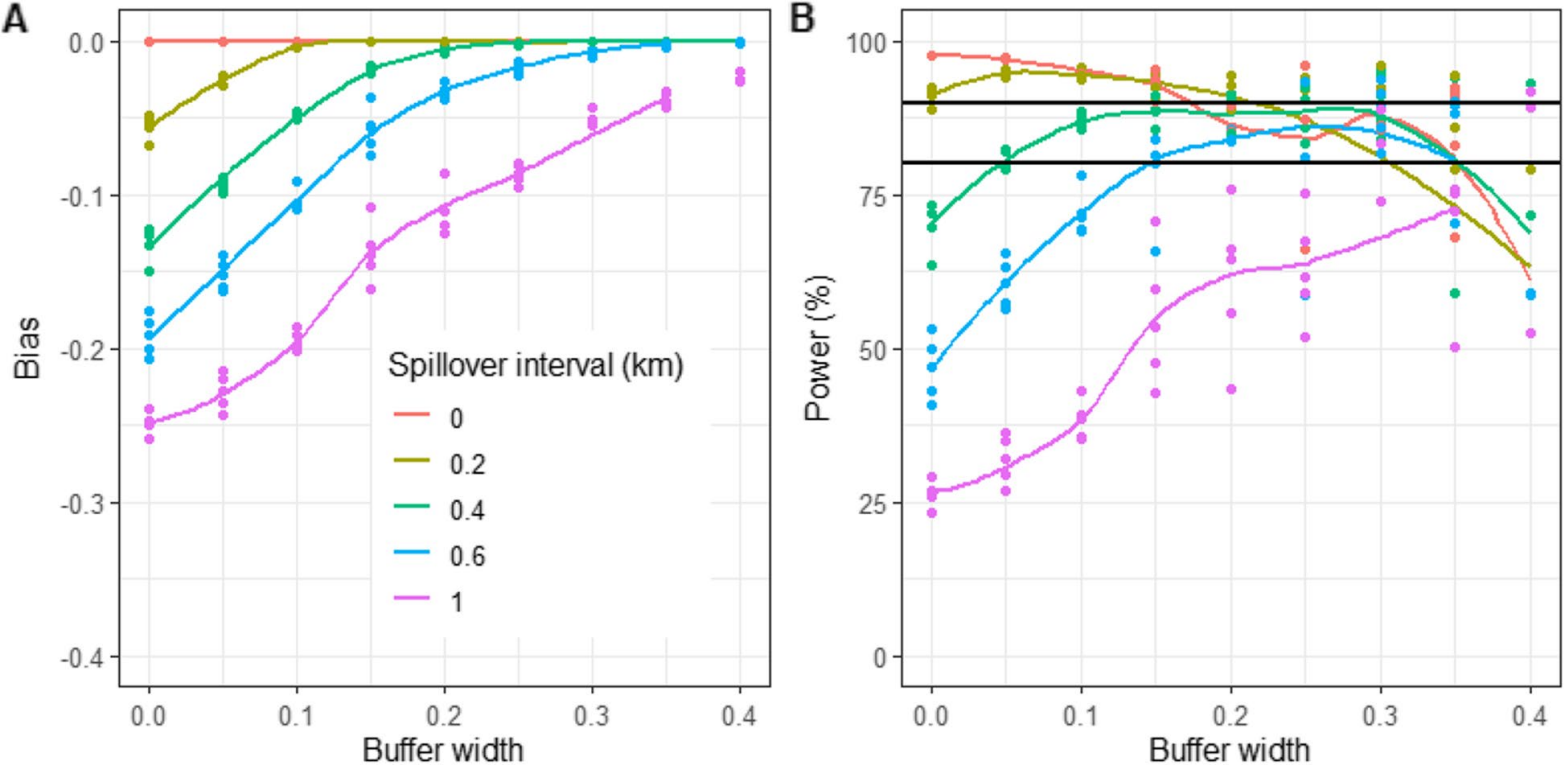
Bias and Power in trials with buffer zones. **A** Bias estimated using Eq. (15) (empirical). **B** Corresponding calculated power. The points correspond to five different randomisations for each combination of spillover interval and buffer width. The lines correspond to loess regressions fitted through the bias or power calculated for the five replicates. An efficacy of ${{E}}=0.4$ was used throughout

A buffer zone might be used to exclude from the analysis the data points closest to the boundary between arms (note that this is different from excluding the margins of the clusters irrespective of the arm of the adjoining cluster, which would exclude approximately twice as many points). An increase in the buffer width leads to a decrease in absolute bias (Fig. 5). If the spillover interval is broad, this results in an increase in power until buffer widths of about 200 m or more are reached. In contrast, with narrow spillover intervals, the power falls as more locations are excluded with the increase in buffer width. With buffer width greater than 200 m most of the data points have been excluded and the analysis depends on which points remain in the core.

## Discussion

The diffusion model provides a parsimonious way of analysing mosquito movement in a CRT. This can be used to allow for spillover in formal calculations of power and sample size, adding the need for just two additional quantities: the distances between sampled points and the discordant arm of the trial, and the anticipated distribution of mosquito movement. This provides, for the first time, an analytical approach for calculating the likely bias caused by spillover, the power of different designs, and the required sample size, both in terms of the amount of data per cluster and the number of clusters. A vignette showing how these calculations can be used in trial design is available at https://thomasasmith.github.io/articles/Usecase10.html.

Just as conventional power and sample size calculations required specification of the distribution of inter-cluster means of the outcome, these calculations require explicit definition of the distribution of how far mosquitoes are displaced between the locations where the intervention may act, and where the outcome is measured. This distribution is here summarized by either ${\sigma }_{m}$ or $s$ (Table 1), but the properties of the normal curve mean that other summary statistics can be readily calculated. For instance, the mean absolute displacement which perhaps best corresponds to the lay notion of how far a mosquito moves is $\sqrt{\frac{2}{\pi }}{\sigma }_{m}=\frac{s}{\sqrt{2\pi }{\Phi }^{-1}(0.975)}\sim \frac{s}{4.9}$. In the example, the analysis provides reassurance that the trial has adequate power to detect intervention effects, at least for cases where the efficacy is 0.4 or greater, and where the mean displacement of the mosquitoes is a few hundred meters at most.

The retention (or even enhancement) of the power, even if much of the data falls in buffer zones and hence excluded from analysis, strongly implies that including sufficient clusters is the key to achieving adequate power. Only when clusters are completely absorbed into the buffers is substantial power lost, since clusters contributing small numbers of datapoints remain informative. However, if most of the area is relegated to the buffers there is a danger of unrepresentativeness of the residual core. This is particularly apposite in the example trial of Zahouli et al. [12], where a few locations outside any of the randomized pixels are included in the cluster to which they are nearest. These locations are unlikely to be in the buffer zones. Investigators should think carefully before discarding most of their data and the algorithm for buffer specification should be documented in the protocol.

Spillover in vector control trials can result from movements of either mosquitoes or humans, or from spread of commodities or practices outside the assigned clusters. CRTs with entomological outcomes of interventions against *Aedes aegypti* in low-income urban settings have also found spillover decreasing over distances of a few tens to hundreds of meters [15, 16] consistent with the main explanation being short-range mosquito movement. The mean flight distance of *Aedes aegypti* is generally little more than 100 m [4] so displacements of this order were assumed for the power calculations presented here for the example trial of Zahouli et al. [12]. In such trials some of the spillover might also be due to geographical spread of larval source management techniques beyond the intervention clusters by word-of-mouth. Human movement is particularly relevant with day-biting mosquitoes like *Aedes* where people commute to remote workplaces and schools [17]. The diffusion model is consequently not appropriate for epidemiological trials of Aedes-borne viruses because patterns of human migration and commuting are poorly described by diffusion. Such movement can be modelled directly using questionnaire-based records of where people spend their time (see for example [18]). Where survey data are unavailable human movement models may be parameterized using a variety of sources, such as transport networks or mobile phone data [19]. In public health applications gravity models or radiation models are often used to represent human mobility [20] rather than diffusion.

The method is applicable to malaria CRTs with either entomological or epidemiological outcomes. In malaria CRTs, spillover can more confidently be attributed to mosquito movement because transmission occurs mainly at night when the positions of almost all the human hosts correspond to the recorded geolocations. Analyses of trials with adjacent clusters confirm that spillover decreases with distance from the boundary between trial arms over hundreds of meters [9, 21–25]. In some trials [21, 22] spillover measured with some outcomes appears to be somewhat asymmetric. This is to be expected if the measured outcome is a downstream effect that is not proportionate to exposure to mosquitoes. In particular, at high transmission, malaria morbidity and mortality incidence are concave functions of exposure to infective mosquitoes [26]. The diffusion model could be extended to allow for such exposure–response relationships, but this would be at the expense of significant complications.

The model is a deliberate simplification, treating such complications as noise. In areas with distinct breeding habitats, or strong environmental gradients, it might be feasible to improve it to allow for non-isotropic movement or deviations from normality. For instance, in The Gambia, dispersal of Anopheles is almost entirely from well defined breeding sites along the river and has been described by specific non-linear regressions [27]. This is an exception. Estimating the distribution of displacement distances of mosquitoes is more challenging than studying human movement, and most analyses of mosquito movement in the field are based on mark release recapture studies [8, 28]. The reported data on distances between release locations and the recapture sites are broadly coherent with the scale of spillover in CRTs but provide poor estimates of the mean of the distribution of displacement distances and cannot be used to evaluate the normal approximation. In practice, trialists rarely have available data or time to develop bespoke models for their sites during the planning phase of trials. It is an advantage that the model adds only one additional unknown parameter (that can be addressed with sensitivity analyses) to power calculations.

At the same time, the ubiquity of diffusion-like processes in nature means the models presented here might well be adaptable for modelling spillover in both CRTs and unclustered randomized trials of other interventions.

## Summary

Standard power calculations do not allow for spillover in CRTs, and usual practice, especially with malaria trials, has been to try to avoid spillover by designing trials assigning large clusters, often with wide buffer zones. The diffusion model provides a straightforward way of extending power formulae to allow for spillover and of predicting the bias that might be expected with different trial designs. The calculations highlight that in much of the parameter space that was considered, trials with many relatively small clusters would be more powerful, and spillover bias is not great.

## Data Availability

The example dataset forms part of the data of the CRT of Zahouli et al. [12], and is subject to the same access conditions as the other trial data. Software for implementing the method is available in the R package CRTspat, which is available on CRAN (https://cran.r-project.org/).

## Abbreviations

CRT: Cluster randomized trial
GEE: Generalized estimating equation
cdf: Cumulative distribution function
pdf: Probability density function
